# *Schinus molle* Resin Essential Oil as Potent Bioinsecticide Against *Tribolium castaneum*: Chemical Profile, *In Vitro* Acetylcholinesterase Inhibition, DFT Calculation and Molecular Docking Analysis

**DOI:** 10.3390/biom14111464

**Published:** 2024-11-18

**Authors:** Wiem Baccari, Ilyes Saidi, Achref Jebnouni, Safa Teka, Sayda Osman, Awatif Mansoor Alrasheeday, Nuzaiha Mohamed, Mabrouka El Oudi, Hichem Ben Jannet

**Affiliations:** 1 Laboratory of Heterocyclic Chemistry, Natural Products and Reactivity (LR11ES39), Medicinal Chemistry and Natural Products Team, Faculty of Science of Monastir, University of Monastir, Avenue of Environment, Monastir 5019, Tunisia; wiem.baccari9@gmail.com (W.B.); sisoelyesaidi@live.fr (I.S.); 2 Medical Surgical Nursing Department, College of Nursing, University of Hai’l, Hai’l 55476, Saudi Arabia; achrefjebnouni1@gmail.com; 3 Department of Chemistry, College of Science, University of Hai’l, Hai’l 55476, Saudi Arabia; safateka@gmail.com (S.T.); doc_mo@ymail.com (M.E.O.); 4 Department of Public Health, College of Public Health and Health Informatics, University of Hai’l, Hai’l 55476, Saudi Arabia; saydaosman91@gmail.com (S.O.); dr.nzeeha@gmail.com (N.M.); 5 Nursing Administration Department, College of Nursing, University of Hai’l, Hai’l 55476, Saudi Arabia; a.alrasheeday@uoh.edu.sa

**Keywords:** *Schinus molle* L., resin essential oil, insecticidal activity, *Tribolium castaneum* (Herbst), acetylcholinesterase inhibition, molecular docking, DFT calculation

## Abstract

Plants offer a bountiful source of natural pest control solutions through their essential oils. This research introduces and analyzes an eco-friendly natural essential oil for red flour beetle control. Therefore, the current study was included to show the chemical profile and the insecticidal efficacy of resin essential oil (REO) and its fractions (F_1–3_), resulting from chromatographic separation, from the plant *Schinus molle* against *Tribolium castaneum* adults. The trunk bark resin essential oil and its fractions’ composition were analyzed by GC-MS. Overall, 33 constituents with 98.3% of the total EO composition were identified. REO and F_1–3_ displayed impressive repellent properties at a concentration of 0.12 µL/cm^2^. After 120 min of exposure, repellency ranged from 73.3% to a remarkable 96.7%. They also exhibited noteworthy fumigant properties, with median lethal doses of LD_50_ = 120.6–160.8 μL/L. The fractions F_1_ and F_3_ showed the most notable topical toxicity at a concentration of 10%, with LD_50_ values of 8.6% and 5.6%, respectively. Fractions F_3_ and F_2_ demonstrated the most effective inhibition of acetylcholinesterase (AChE) activity, providing insight into their insecticidal mechanisms. The in silico molecular docking and DFT studies corroborate the results of in vitro tests performed to identify new insecticide products derived from natural sources.

## 1. Introduction

Essential oils have been used for centuries for their diverse therapeutic properties and applications. These natural extracts, derived from plants, are known for their aromatic and medicinal qualities and are extensively utilized in aromatherapy, cosmetics, and traditional medicine [1,2,3]. Most essential oils are composed of a diverse array of volatile organic compounds, predominantly featuring a complex mixture of monoterpenes, sesquiterpenes, alcohols, esters, aldehydes, and oxides. These compounds are typically derived from isoprene units, which are formed from a 5-carbon (C_5_) base [1,4]. Essential oils possess antifungal [5,6], antibacterial [5], antiviral [7], antioxidant [8,9], cytotoxic [10,11], anti-α-amylase, anti-butyrylcholinesterase [9], and anti-acetylcholinesterase [8,9,12] properties, making them valuable in the pharmaceutical and food industries. Additionally, they have been explored as natural pesticides due to their insecticidal and repellent activities [13,14], offering an eco-friendly alternative to synthetic chemicals. Their potential in integrated pest management is particularly significant as they provide a sustainable solution to control pests without harming the environment.

With the global population soaring towards 9.1 billion by 2050, humanity faces a colossal challenge: producing 70% more food to keep everyone fed [15]. Developing nations, where hunger lingers even now, are poised to fuel the bulk of population growth. This surge strains already fragile food systems as urbanization, climate woes, and non-food crops compete for the land. However, post-harvest losses of agricultural products, particularly staples like grain and maize, cast a long shadow on global food security, a problem often shrouded in neglect [16]. According to the FAO, insects are estimated to be responsible for up to 30% of global post-harvest losses, significantly influencing food security [17]. Furthermore, millions of USD worth of stored grains lose their market value each year due to insect infestations, influencing food security and economies.

The red flour beetle (*Tribolium castaneum*), a devastating member of the Tenebrionidae family, plagues stored products across the globe. Flour heavily infested with *T. castaneum* emits a pungent odor and leaves a moldy aftertaste. The beetles’ bitter quinone secretions compromise baking quality [18]. Phosphine fumigation, organophosphate, and pyrethroid contact insecticides constitute the primary chemical control measures for *T. castaneum* and other insect pests in stored products around the world [19]. Faced with growing environmental concerns, exploring botanical insecticides as part of integrated pest control offers a compelling, green alternative [20]. Plant-derived essential oils, rich in sesquiterpenes, are emerging as a promising alternative for insect control in stored products [13,21,22,23,24,25]. One of these plants, *Schinus molle* L., recognized for its diverse array of bioactive compounds, has demonstrated potential for various medicinal and culinary applications, including antitumoral, antibacterial, antifungal, antioxidant, anti-tuberculosis, anti-inflammatory, antiviral, anti-spasmodic, and analgesic properties [26,27]. These activities reflect the plants’ natural defense mechanisms via essential oils [6,9,20]. The insecticidal and repellent activities of *S. molle* extracts and essential oils have been evaluated against various insect species in several published studies [20,28,29,30,31]. The activity exerted by EO constituents on numerous insects involves neurotoxic effects through various mechanisms, most notably GABA and octopamine synapses, as well as acetylcholinesterase inhibition. This neurotoxicity mechanism targets nervous system receptors. A critical enzyme targeted by many insecticides in the nervous system is acetylcholinesterase [32].

Surprisingly, the potential of *S. molle* resin EO as an insecticide has not yet been investigated. This paper breaks new ground, unveiling the hitherto unknown insecticidal and repellent properties of this EO against *T. castaneum* adults. The chemical composition of this EO and its fractions was also determined by GC-MS analysis. To uncover the possible mechanism of action of *S. molle* REO on *T. castaneum* adults, the acetylcholinesterase inhibitory potential was evaluated. An in silico molecular docking study was conducted to gain insight into the affinity of the major compounds for the target acetylcholinesterase enzyme. Using Density Functional Theory (DFT), a detailed theoretical study was performed to evaluate the molecular reactivity and stability of these compounds.

## 2. Materials and Methods

### 2.1. Plant Material

In June 2021, the trunk bark resins (24 g) of *Schinus molle* L. were collected in the garden of the Faculty of Sciences of Monastir, governorate of Monastir (Latitude: 35°45′, Longitude: 10°48′). Plant material with the number SM-21 is deposited in the Laboratory of Heterocyclic Chemistry, Natural Products and Reactivity (LR11ES39), Faculty of Sciences of Monastir, Tunisia.

### 2.2. Extraction and Fractionation of the Essential Oil

The fresh resins (24 g) were subjected to hydrodistillation for 6 h on a Clevenger apparatus. The obtained EO was decanted, dried (with Na_2_SO_4_ (VWR International, Leuven, Belgium), weighed, and conserved at 4–5 °C in the dark in a freezer until testing.

A determined quantity of crude essential oil (m = 2.867 g) from the resin of *S. molle* was chromatographed using silica gel column chromatography eluted with hexane (Fisher scientific, Loughborough, UK) and progressively enriched with ethyl acetate (Fisher scientific, Loughborough, UK) (10 to 50% EtOAc). The collected fractions were checked by TLC plates (Merck KGaA, Darmstadt, Germany) and those with a similar profile were mixed to give three main fractions (F_1_ = 1.6 g, F_2_ = 0.14 g, and F_3_ = 0.185 g).

### 2.3. Chromatographic Analysis

The composition of the EO was analyzed using Gas Chromatography-Electron Impact Mass Spectrometry (GC-EIMS). Analyses were performed with a Varian CP-3800 gas chromatograph (Agilent Technologies Inc., Santa Clara, CA, USA) using an Agilent DB-5 capillary column (Agilent Technologies Inc., Santa Clara, CA, USA), dimensions: 30 m, 0.25 mm, 0.25 μm film thickness and coupled with a Varian Saturn 2000 ion-trap mass detector (Agilent Technologies Inc., Santa Clara, CA, USA).

The operational context was characterized as follows: injector temperature 220 °C; transfer line temperature 240 °C; oven temperature, 60 to 240 °C at 3 °C/min; carrier gas helium at 1 mL/min flow. Injection of 1 µL (5% HPLC grade *n*-hexane) into the GC (split ratio 1:30). The acquisition parameters were as follows: full scan (30–300 *m*/*z* range); scan time of 1.0 s. To identify the components, we compared the retention times of our four tested samples with those of pure, authentic samples. Specifically, we looked at their linear retention indices relative to a series of *n*-hydrocarbons (C_5_–C_25_). Beyond standard comparisons, we utilized computer matching against a commercial (NIST, 2014) and a mass spectral library, built up from pure components of commercial essential oils of known composition and MS literature data NIST/EPA/NIH Mass Spectral Library, respectively [33].

### 2.4. Insecticide Activity

#### 2.4.1. Insects Rearing

The *Tribolium castaneum* adults used in this study were raised on a diet consisting of wheat flour and maize. The insects were kept in a controlled environment at 27 °C (with slight variations), 60% humidity, and a 16-h light/8-h dark cycle.

#### 2.4.2. Repellent Activity Bioassay

Following the area preference method of McDonald et al. (1970) [34], this study evaluated the repellency of an essential oil (EO) and its fractions (F_1_–F_3_) against *T. castaneum* adults. Whatman filter paper discs (9 cm diameter) were bisected, with one half treated with 12 µL of phytochemical solution diluted in 600 µL acetone and the other half serving as an acetone control of acetone (200 µL). Twenty insects were introduced to the center of the combined disc and their numbers on each half were counted at 15, 30, 60, and 120 min. Repellency percentages were calculated based on the formula provided and categorized using the McDonald et al. (1970) scale [34].

PR(%) = [(Nc − Nt)/(Nc + Nt)] × 100(1)

Nc: Number of pests present on the negative control semicircle. Nt: the treated semicircle was counted.

#### 2.4.3. Fumigant Activity Bioassay

The fumigant potential of essential oil (EO) and its fractions (F_1_–F_3_) against *T. castaneum* adults was assessed using a 40 mL glass vial bioassay. Three-centimeter filter paper disks (Whatman No. 1) were saturated with different concentrations of the test samples and placed tightly on the underside of the vial lid. Ten insects were then introduced into each vial, which was promptly sealed. The fumigation test was conducted at 27 °C ± 2 °C for 24 h, with three replicate vials per dose. Mortality percentages were determined by scoring immobile insects unresponsive to gentle antennal contact with a paintbrush [13].

#### 2.4.4. Contact Toxicity Bioassay

A topical application bioassay was employed to assess the contact toxicity of *S. molle* resins EO and its fractions against *T. castaneum* adults. To begin, 1 µL of each test sample, prepared in three different concentrations (1%, 5%, and 10%) using acetone dilutions, was precisely deposited on the pronotum of insects. Following solvent evaporation, ten insects were introduced into each Petri dish (Ø: 9 cm). A control group was treated with acetone alone. The bioassay was conducted with three replicates per dose. Mortality rates were determined after 24 h of exposure, with the absence of antennal or leg movement defining an insect as dead [13].

### 2.5. Acetylcholinesterase Inhibitory Assay

The anti-acetylcholinesterase potential of *S. molle* EO and its fractions was assessed using a slightly modified version of the Ellman method [35]. All samples were first dissolved in DMSO and then diluted with a buffer (0.1 M of sodium phosphate (Na_3_PO_4_) (pH = 8.0)), ensuring that the final DMSO concentration in the mixture did not exceed 1%. Then, 25  μL of each pre-prepared sample, 50 μL of the buffer solution, 25 μL of AChE enzyme, and 125 μL of DTNB (dithiobisnitrobenzoic acid; 3 mM; pH = 8.1) were combined, then incubated in a 96-well microplate at 25 °C for a quarter of an hour. Subsequently, 25 μL of acetylthiocholine iodide (ACTI) solution was introduced into the test wells containing the mixture, which was then incubated under the same conditions mentioned above. After incubation, the absorbance was recorded at 410 nm. The negative control included all components mentioned above except the tested samples. Galantamine served as the positive control in the experiment.

### 2.6. Molecular Docking Procedure

The chemical skeletons of galantamine and the eight main constituents (>10%) from the *S. molle* REO and their fractions F_1–3_ were generated and optimized using ACD; 3D viewer software (ACD/Labs 2017.2.1). The receptor protein, namely the acetylcholinesterase enzyme (AChE) from *T. castaneum*, was obtained from “www.ncbi.nlm.nih.gov” as an amino acid sequence. The downloaded sequence from the FASTA section was modeled for a 3D quaternary structure using the platform Swiss-model (https://swissmodel.expasy.org/ (accessed on 21 May 2024)). Based on the alignment of the sequence, which was confirmed for accuracy, the crystal structure was chosen as an appropriate template for protein modeling. The molecular docking process was performed using the software AutoDock Vina (v1.1.2) [36]. For the visual representation of the molecule-enzyme bindings, the BIOVIA DSV (2017, version 17.2.0.16349) (Discovery Studio Visualizer, Dassault Systèmes BIOVIA: San Diego, CA, USA) was employed.

### 2.7. Computational Details (DFT Studies)

DFT: Density Functional Theory calculations for the eight compounds from the *S. molle* REO and their fractions F_1–3_ including (*E*)-β-caryophyllene (**2**), elemol (**10**), germacrene B (**11**), caryophyllene oxide (**13**), β-eudesmol (**22**), α-eudesmol (**23**), elemyl acetate (**27**), and (*E*,*E*)-farnesyl acetate (**33**) were drawn using ChemDraw 21.0.0 and Chem3D 21.0.0. In conjunction with the “6-31G (d, p)” basis set, the DFT-B3LYP modelling method was employed to optimize the geometries of the selected compounds [6,37,38]. All calculations were carried out utilizing the Gaussian 09W program [39] and the GaussView 6.0 visualization program [40].

### 2.8. Statistical Analysis

All statistical analyses were conducted using SPSS software (Version 23.0) [41]. To evaluate differences between means, Duncan’s multiple range test was applied at a significance level of *p* < 0.05. Mortality data were adjusted using Abbott’s correction formula [42]. LD_50_ values were calculated via probit analysis, utilizing data from all replicates [43].

## 3. Results and Discussion

### 3.1. Chemical Composition

**Figure 1:**
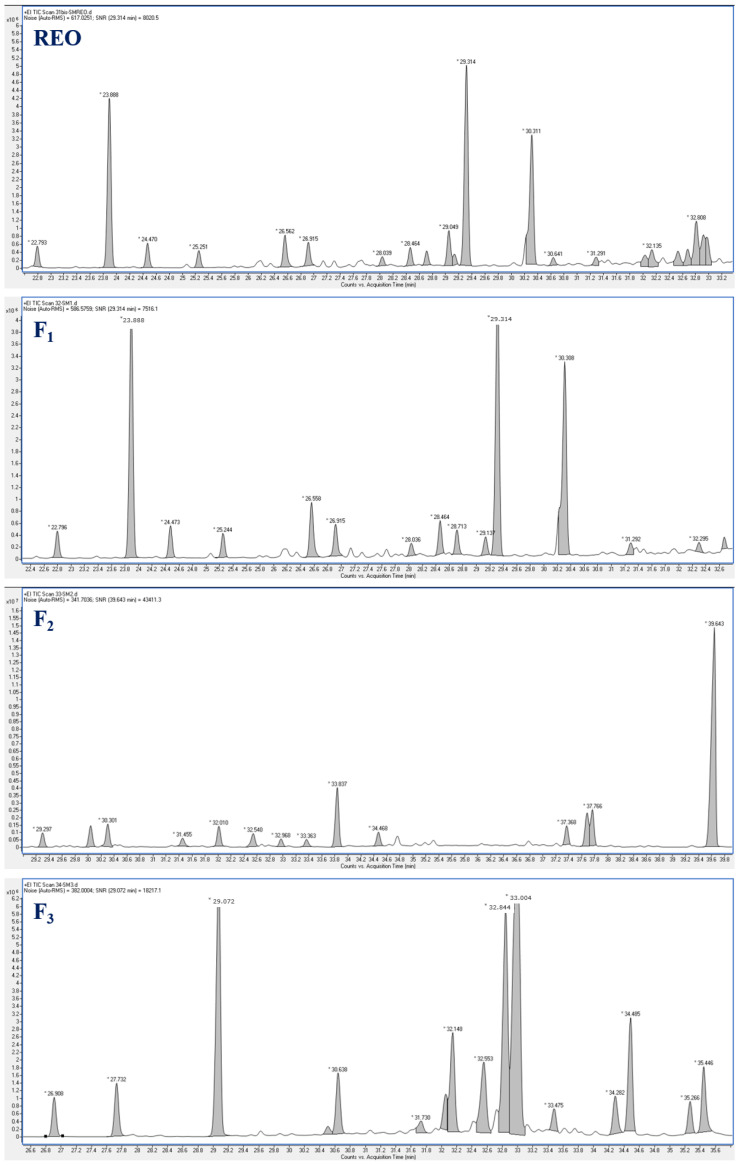
GC-MS chromatograms of resin essential oil of *Schinus molle* (REO) and its fractions (F_1–3_). * Retention time of identified component.

Hydrodistillation of fresh resin *S. molle* yielded 11.95% (*w*/*w*), with yellow colored EO. The crude EO was fractionated into three fractions using column chromatography. Table 1 presents the detailed chemical composition of the studied EO and its fractions, as analyzed by GC-MS. GC-MS chromatograms of resin essential oil of *Schinus molle* (REO) and its fractions (F_1–3_) are shown in Figure 1.

Guided by the dual objectives of elucidating minor constituents and isolating insecticidal components, the study employed a targeted chromatographic simplification approach. This fractionation process successfully concentrated specific insecticidal compounds (sesquiterpene hydrocarbons or oxygenated sesquiterpenes) in particular fractions, facilitating their subsequent identification and bioactivity evaluation.

Analysis of the resin REO and its fractions F_1–3_ led to the identification of 22, 13, 13, and 12 compounds, respectively, representing 98.3, 96.3, 93.6, and 95.1% (Table 1). The raw EO and the three fractions predominantly contained (*E*)-β-caryophyllene (**2**), elemol (**10**), germacrene B (**11**), caryophyllene oxide (**13**), β-eudesmol (**22**), α-eudesmol (**23**), elemyl acetate (**27**), and (*E*,*E*)-farnesyl acetate (**33**).

Both the resin of *S. molle* and the first fraction (F_1_) consisted chiefly of sesquiterpene hydrocarbons (49.9 and 64.9%, respectively). Table 1 shows their distribution and indicates that these two samples (REO and F_1_) were rich in components such as (*E*)-β-caryophyllene (**2**) (REO: 17.7%; F_1_: 24.2%) and germacrene B (**11**) (REO: 21.4%; F_1_: 26.5%). It has been noted that the percentage of hydrocarbon sesquiterpenes decreased during the fraction process from 64.9 to 0.0%. However, the second (F_2_) and third (F_3_) fractions were mainly composed of oxygenated sesquiterpenes (91.0% and 95.1%, respectively). The main detected oxygenated sesquiterpenes were caryophyllene oxide (**13**) (REO: 15.1%; F_1_: 21.7%), β-eudesmol (**22**) (F_3_: 16.0%), α-eudesmol (**23**) (F_3_: 26.8%), elemyl acetate (**27**) (F_2_: 10.9%), and (*E*,*E*)-farnesyl acetate (**33**) (F_2_: 48.3%).

While major essential oil compounds defied pure isolation via column chromatography, the technique facilitated concentration of specific products and identification of minor ones, such as cubebol (**7**), germacrene D-4-ol (**12**), humulane-1,6-dien-3-ol (**16**), caryophylla-4(12),8(13-dien-5-ol (**19**), *epi*-γ-eudesmol (**25**), bulnesol (**26**), (*Z*,*E*)-farnesol (**28**), juniper camphor (**29**), (*E*,*E*)-farnesol (**30**), β-eudesmyl acetate (**31**), and γ-eudesmyl acetate (**32**).

Despite a comprehensive literature search, only one study was found on the chemical composition of *S. molle* resin essential oil. This underscores the limited research available in this area. In fact, the composition of the Tunisian *S. molle* resin essential oil presents several differences compared to the same oil isolated from other countries. In our REO, no monoterpenes were detected, while hydrocarbons and oxygenated sesquiterpenes dominated by percentages of 49.9% and 48.4%, respectively. Sesquiterpenes and monoterpenes hydrocarbons (81.2 and 3.2%, respectively) constituted the most abundant fractions of the oil collected in Turkey [44]. Unlike our oil, which is rich in oxygenated sesquiterpenes, the Turkish resin’s essential oil harbors only a trace (0.7%) of these compounds [44]. Apart from a few constituents, such as (*E*)-β-caryophyllene (17.7% in Tunisian resin, 18.3% in Turkish resin) and germacrene B (21.4% in Tunisian resin, 20.2% in Turkish resin), the chemical profiles of the two oils show little similarity.

### 3.2. Insecticidal Activity

#### 3.2.1. Repellent Activity

The repellent efficacy of the *S. molle* resin EO toward *T. castaneum* adults was determined by the McDonald method. Table 2 displays the repellency percentages of the EO and its fractions, tested at a concentration value of 0.12 µL/cm^2^ over four varied exposure times.

The study found that *S. molle* resin essential oil exhibited strong repellent activity against *T. castaneum* adults, reaching 76.7 ± 5.8% repellency after 120 min of exposure. This classified it as a highly effective (Class IV) repellent agent. While the crude essential oil (EO) showed good repellent activity, chromatographic fractionation revealed even stronger potential within its components. Notably, all fractions except the second (F_2_) displayed enhanced bioactivity, exceeding 73.3 ± 5.8% repellent effect. This finding highlights the value of fractionation for isolating and concentrating active compounds. In fact, F_3_ demonstrated a highly effective repellent effect early on, achieving 90.0 ± 10.0% repellency within the first hour of exposure.

A previous study has been conducted specifically on the repellent properties of Saudi Arabian *S. molle* essential oil (fruit) at a concentration 10,000 µL (85.11%) towards the storage pest *T. castaneum* [29]. This finding suggests potential for the EO of *S. molle* to be effective against *T. castaneum*. The hexane extracts from both the fruits and leaves of *S. molle* also demonstrated repellent activity against larvae of *Cydia pomonella* and *Triatoma infestans* [45]. In another published study, Belhoussaine et al. (2022) tested the repellent potential of *S. molle* fruit and leaf EOs against adults of *Sitophilus oryzae*, revealing a moderately repulsive effect (Class II) with repellency percentages of 28.18% and 21.52%, respectively, at various concentrations [46]. Laoudi et al. (2023) found that after 30 min of exposure, at the highest tested concentration, the leaf EO of the Algerian *S. molle* was classified as class III (moderately repellent) against *Oryzaephilus surinamensis*, with a percentage of 55.62% [47].

The remarkable repellent effect of the tested EO and its fractions can be linked to their rich blend of terpenes, which are well-established anti-insect compounds, particularly in terms of repellency [13,48,49]. The observed strong repellent effect of the REO and F_1–3_ likely comes from the main identified compounds. These compounds all belong to the category of sesquiterpenes (hydrocarbons and oxygenated), which are well-known for their effectiveness as insect repellents. In fact, (*E*)-β-caryophyllene (**2**) (REO: 17.7%; F_1_: 24.2%) is among the main compounds responsible for the important repellent properties against *Aedes aegypti* [50]. Furthermore, caryophyllene oxide (**13**) (REO: 15.1%; F_1_: 21.7%) is a common component found in many essential oils (EOs) previously studied for their insect repellent properties [13,25,51]. Additionally, the key role of elemol (**10**) (REO: 3.6%; F_1_: 1.8%; F_3_: 16.6%) as repellent substance has been documented in several studies. As an example, this compound demonstrated strong repellency (100%) at 155 nmole elemol/cm^2^ against *Ixodes scapularis* nymphs from the start of the exposure time (15 min) [52]. On the other hand, β-Eudesmol (**22**) (REO: 5.5%; F_3_: 16.0%) isolated from the aerial part EO of the Chinese *Epimedium pubescens* showed an interesting repellent effect against *T. castaneum* (RP = 78.0% at concentration of 16.0 nL/cm^2^) and *Liposcelis bostrychophila* (RP = 95.0% at concentration of 4.3 nL/cm^2^) after two hours [53]. Nevertheless, the synergistic effect of some minor compounds cannot be overlooked.

The present tested fractions F_1_ and F_3_ showed the most significant repellent results, with repellency values of 86.7% and 96.7%, respectively, after 2 h of exposure. The observed difference in the repellency results between these two fractions can be attributed to the fluctuating proportions of certain active compounds, such as *epi*-cubebol (**5**) and elemol (**10**). Furthermore, some constituents exclusively identified in fraction F_3_, such as cubebol (**7**) (3.1%), bulnesol (**26**) (1.2%), (*Z*,*E*)-farnesol (**28**) (2.5%), and (*E*,*E*)-farnesol (**30**), (3.9%) may contribute partially to the enhancement of the activity. In fact, farnesol and its isomers have been extensively documented for their repellent properties [54].

#### 3.2.2. Fumigation Test

**Figure 2:**
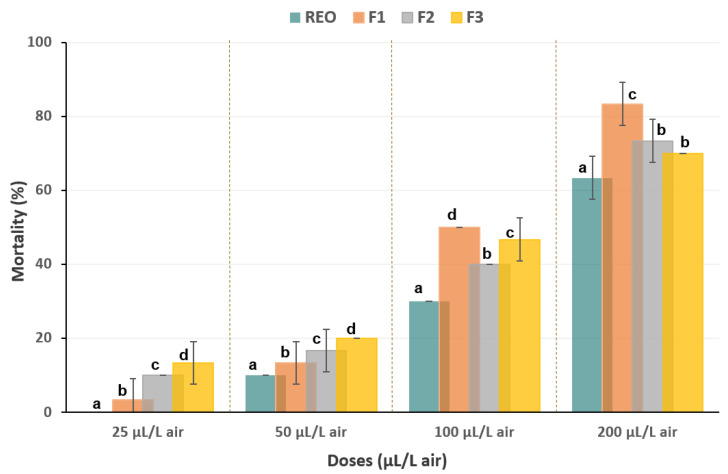
Percentage of mortality of *Tribolium castaneum* adults after 24 h of exposure to various concentrations of the resin essential oil (REO) of *Schinus molle* tree and its fractions (F_1–3_) using the fumigation bioassay. The same letter above the bars indicates that there is no statistically significant difference at a *p*-value less than 0.05.

*Schinus molle* resin essential oils (EOs) are effective fumigants against the red flour beetle (*T. castaneum*) due to their volatility, which allows them to reach and kill insects in hidden spaces. This fumigant effect complements the repellent activity of the EOs. Fumigation is the most widely applied method for post-harvest beetle control [55], and the results of this study are shown in Figure 2.

Following 24 h of exposure to four gradually increasing concentrations of the essential oils, varying results were observed. Only the three fractions (F_1–3_) demonstrated limited toxicity at the lowest concentration (25 μL/L air), with mortality not exceeding 14%. The effectiveness of the tested oils increased with concentration.

The essential oil (EO) and its fractions showed significant fumigant toxicity with slight difference in mortality rates at the highest concentration (200 µL/L air). While EO reached 63.3%, F_1_, F_2_, and F_3_ achieved even higher rates of 83.3%, 73.3%, and 70.0%, respectively (Figure 2). This significant increase in mortality rates highlights the strong dose-dependent effect of the essential oil (EO) and its fractions on pest control. The lethal doses of the EO and F_1–3_ were estimated to be 160.8, 120.6, 134.4, and 131.6 μL/L air, respectively.

In fact, fractionation proved to be a powerful tool for enhancing the potency of the essential oil against pests. Compared to the whole oil, the separated fractions (obtained through chromatography) demonstrated significantly higher effectiveness. This suggests that some fractions carry particularly concentrated active ingredients.

The fumigant toxicity of *S. molle* fruit and leaf EOs from Saudi Arabia was reported on *T. castaneum* with LC_50_ values of 286.1 and 361.1 μL, respectively [29]. Recently, the EO extracted from the fresh leaves of the same plant also showed fumigant efficacy on *Oryzaephilus surinamensis* strain with LD_50_ = 72.5 μL/L at concentrations ranging from 40 μL/L to 100 μL/L [47].

The fast-acting nature of EOs, due to their volatility, likely contributes significantly to the effectiveness of fumigation when compared to slower-acting residual contact methods that require penetration of the insect’s exoskeleton [56]. The fumigant action of EOs relies heavily on their diverse chemical profiles, which are rich in terpenes such as monoterpenes and sequiterpenes. Numerous studies have documented the potent insecticidal activity of these terpene classes, especially in fumigant applications [57,58,59]. It is important to consider the potential fumigant effect of major compounds like (*E*)-β-caryophyllene (**2**) and caryophyllene oxide (**13**). These compounds have been shown to have such properties in previous studies by Born et al. (2022) [60] and Wu et al. (2021) [58]. Indeed, among various pure products belonging to the terpenoid family, the tested mite (*Tetranychus urticae*) was more sensitive to (*E*)-β-caryophyllene, with an LC_50_ value of 0.05 μL/L of air at concentrations ranging from 3.2 × 10^−4^ to 16.0 μL/L air [60]. In the other study, caryophyllene oxide showed good fumigant potential against *T. castaneum* after 48 h (LC_50_ = 97.72 μL/L of air) and 72 h (LC_50_ = 65.22 μL/L of air) at concentrations ranging from 16 to 256 μL/L of air. In addition, elemol (**10**) (F_3_: 16.6%), a widespread constituent of many EOs outlined in several previous papers, has demonstrated insect fumigant properties [14,61]. On the other hand, EOs containing germacrene isomers, particularly germacrene B (**11**) (EO = 21.4%; F_1_ = 26.5%) as one of their main constituents, also demonstrated a good fumigant effect against various insects. For example, the leaf essential oil of *Juniperus polycarpus* was toxic to *T. confusum*, with an LC_50_ value of 368 μL/L air at concentrations ranging from 111 to 611.05 μL/L of air [62]. Similarly, the insecticidal properties of eudesmol compounds such as β-eudesmol (**22**) (F_3_: 16.0%) and α-eudesmol (**23**) (F_3_: 26.8%), have not been extensively studied to date. However, there are some reports indicating that these elements are part of the chemical composition of various EOs with fumigant activity. For example, *S. molle* leaf EO (β-eudesmol: 15.19%) [47] and the aerial part EO of *Drimys winteri* (γ-eudesmol: 11.42%, β-eudesmol: 8.49%, α-eudesmol: 6.39%) demonstrated fumigant efficacy against *Acanthoscelides obtectus* with LD_50_ value of 60.1 µL/L [61].

The findings mentioned above potentially corroborate the results obtained in the present study. However, it is important to consider that while the main constituents may have significant effects against the concerned pests, the overall bioactivity of the EOs typically relies on the cumulative action of its minor and main constituents. Moreover, the synergistic and/or the antagonistic interactions among their components may influence the EO’s efficacy [63].

The reasoning provided above might explain the fumigant potential observed in the EO examined in this study; however, additional research is needed to devise a suitable formulation utilizing this EO. It is crucial to assess the impacts of the individual constituents as well as their synergy, as this could enhance their effectiveness. In this context, the molecular docking study was conducted in the following sections, during which the in silico evaluation of the major compounds was carried out.

#### 3.2.3. Contact Toxicity 

To confirm the insecticidal properties of the essential oil (EO) and the fractions F_1–3_, researchers conducted an additional test using topical application. They assessed the contact toxicity of *S. molle* essential oil against adult *T. castaneum* after one day of treatment, using three different concentrations of each sample.

**Figure 3:**
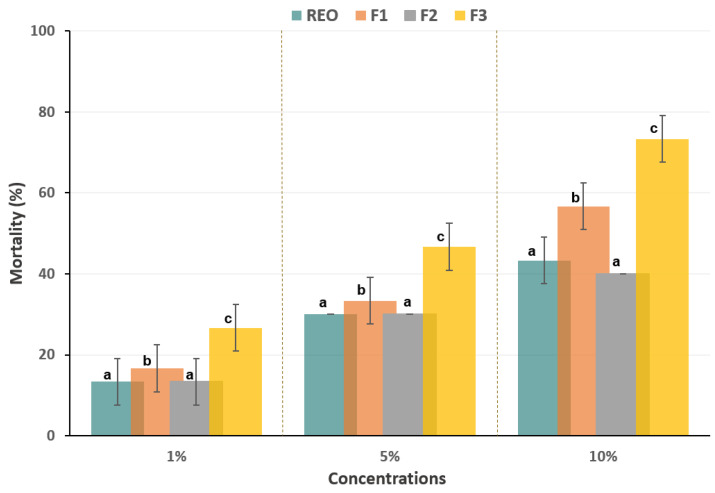
Toxicity of *Tribolium castaneum* treated with the resin essential oil (REO) of *Schinus molle* tree and its fractions (F_1–3_) by topical application bioassay. The same letter above the bars indicates that there is no statistically significant difference at a *p*-value less than 0.05.

Figure 3 shows that all tested samples (REO and F_1–3_) exhibited a dose-dependent response, with the highest mortality rate observed at the 10% concentration. After one day of exposure, the essential oil (EO) reached a mortality rate of 43.3%. Furthermore, fractions F_1_ and F_3_ exhibited more promising activity at the highest concentration (10%), with LD_50_ values of 8.58% and 5.55%, respectively.

The plant family Anacardiaceae, with its wide variety of chemical compounds, was frequently used in tests where substances are applied to topical application [64,65]. In recent years, phytochemicals from the *Schinus* species have attracted significant interest due to their potential as effective insecticides with minimal side effects. This potential was highlighted through studies using contact toxicity bioassays on *Schinus* species. Machado et al. (2019) have studied the topical toxicity of the EO from *S. molle* leaves against bed bug (*Cimex lectularius*) [66], wherein after 7 days this oil showed toxicity (50 ± 10%) at a concentration of 125 μg EO/bed bug [66]. The EO extracted from leaves and mature fruits of the same plant mentioned earlier (*S. molle*) also showed topical efficacy on *Ctenocephalides felis felis* adults with 100% efficacy at doses of 800 µg/cm^2^ (LD_50_ = 353.95 µg/cm^2^) and at 50 µg/cm^2^ (LD_50_ = 12.02 µg/cm^2^), respectively [31].

(*E*)-β-caryophyllene (**2**) and caryophyllene oxide (**13**) are key marker compounds in the Tunisian *S. molle* REO and its first fraction (F_1_), the second most effective tested sample. These compounds may be primarily responsible for their toxicity against *T. castaneum* adults. In a previous study, these chemotypes exhibited contact toxicity against *Megoura japonica* with LD_50_ values of 0.072 and 0.13 μg/adult, respectively [67]. In addition, elemol (**10**) and β-eudesmol (**22**), which are among the primary compounds in the most active fraction (F_3_), have demonstrated their potent insecticidal properties and potential for use in developing effective pest control solutions. These compounds exhibited significant topical toxicity against *Drosophila melanogaster* adults, with LD_50_ values of 0.65 and 2.63 µg/adult, respectively [61,68]. These and other major compounds may play a significant role in the observed toxicity, as their high concentrations can directly affect the target organisms. However, the effects of minor phytochemicals should not be overlooked. Despite their lower concentrations, these minor constituents can contribute to the overall bioactivity through synergistic interactions with major compounds. The topical efficacy of three common essential oil components was tested against *Cimex lectularius*, the properties of the tertiary mixture indicating that their potency cannot be attributed to the unique effect of each component, but rather to the synergetic interactions between the components of the tested mixture [69]. The same principle could explain the efficacy of the present EO and its active fractions, in which a sum of feasible interactions among the main components present could clarify the significant topical effect observed.

### 3.3. Acetylcholinesterase Inhibitory Activity

One of the primary reasons for the toxicity of essential oils (EOs) is their ability to damage the nervous systems of insects. The inhibition of acetylcholinesterase (AChE) is a key mechanism of action for many-studied EOs [70,71]. Indeed, due to this inhibition, acetylcholine accumulates in the synapses, keeping the receptors continuously open and ultimately leading to the organism’s death, such as in the case of insecticidal activity. Given their significant insecticidal potential and the importance of AChE inhibition in pest control, we aimed to investigate whether the insecticidal activity of *S. molle* REO and its fractions was related to the AChE enzyme activity.

Acetylcholinesterase inhibitory activity of tested samples (REO and F_1–3_) is presented in Table 3. With varying degrees of effectiveness, they proved to inhibit the AChE enzyme in vitro. The raw EO, F_1_, F_2_, and F_3_ demonstrated dose-dependent inhibition of AChE, with IC_50_ values of 88.55 μg/mL, 42.77 μg/mL, 15.25 μg/mL, and 13.07 μg/mL, respectively. The latter was the closest to galantamine (IC_50_ = 1.93 μg/mL). The first notable aspect of the obtained results is that the fractions, especially F_2_ and F_3_, showed better results than the raw EO, which, in most cases, aligns with the results of insecticidal activity. On the other hand, the reported results shed light on the synergistic effect of the total constituents of *S. molle* resin EO, which decreased the efficacy and once again highlighted the importance of chromatographic simplification in improving the activity.

The studied EO and its fractions are characterized by 93.6% to 98.3% sesquiterpene compounds, which are primarily responsible for their significant anti-AChE activity [72]. Several essential oils with an absolute predominance of sesquiterpene derivatives have shown to possess potent AChE inhibitory activity. For instance, the leaf essential oil of *Callicarpa candicans*, rich in sesquiterpenes (92.2%) including (*E*)-β-caryophyllene (15.3%) and germacrene B (5.1%), showed significant AChE inhibition with an IC_50_ value of 45.67 μg/mL [73]. In the same research, the authors demonstrated that the sesquiterpene-rich (75.4%) leaf essential oil of *Callicarpa erioclona*, comprising (*E*)-β-caryophyllene (11.1%) and caryophyllene oxide (5.9%), proved to be a good inhibitor of AChE with an IC_50_ value of 28.71 μg/mL. Similarly, a study by Valarezo et al. (2022) has supported this idea by demonstrating the potent AChE inhibition (IC_50_ = 41.51 µg/mL) of leaf EO of *Annona cherimola*, which is rich in sesquiterpene phytochemicals (73.87%) [74].

(*E*)-β-caryophyllene (**2**) has been repeatedly reported to inhibit AChE [75,76]. In 2022, Hung et al. demonstrated that (*E*)-β-caryophyllene significantly inhibited AChE with an IC_50_ value of 89.10 µg/mL. In the same study, they also showed that caryophyllene oxide exhibited anti-AChE potential with lower efficiency (IC_50_ = 320.16 µg/mL). Furthermore, elemol (**10**) (16.6%) and eudesmol derivatives (β-eudesmol (**22**): 16.0% and α-eudesmol (**23**): 26.8%), among the main compounds identified in the most active fraction (F_3_), were found to have potential in inhibiting the AChE enzyme [77,78,79]. However, another study demonstrated that the EO’s main constituents do not always contribute to their activity; minor components often play a significant role instead [80]. EOs consist of complex mixtures of numerous volatile constituents, many of which have not been examined for their anti-AChE activity or for their potential antagonistic or synergistic effects.

### 3.4. Molecular Docking Study

**Figure 4:**
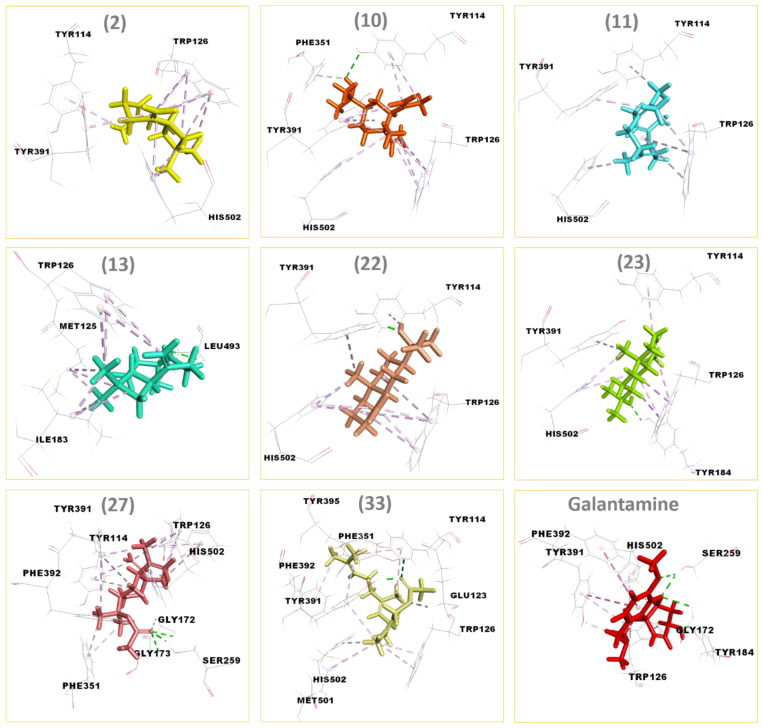
3D representations of the binding modes of *Tribolium castaneum* acetylcholinesterase enzyme with (*E*)-β-caryophyllene (**2**), elemol (**10**), germacrene B (**11**), caryophyllene oxide (**13**), β-eudesmol (**22**), α-eudesmol (**23**), elemyl acetate (**27**), (*E*,*E*)-farnesyl acetate (**33**), and galantamine.

Based on the in vitro anti-acetylcholinesterase activity results obtained in this study, REO and F_1–3_ demonstrated significant inhibitory effects on AChE. To better understand how these compounds interact with the AChE enzyme, a comprehensive molecular docking study was performed for the main identified compounds (>10%) of *S. molle* resin EO and its fractions by using the AutoDock Vina software. The results as binding energy values and interactions details are tabulated in Table 4. The 3D depiction of binding modes of the eight selected compounds, namely (*E*)-β-caryophyllene, elemol, germacrene B, caryophyllene oxide, β-eudesmol, α-eudesmol, elemyl acetate and (*E*,*E*)-farnesyl acetate, all with galantamine, the standard used agent, are shown in Figure 4.

The data in Table 4 reveal that the binding energy values range from −5.7 to −7.8 kcal/mol. A more negative docking score indicates a more stable complex and a greater binding affinity of the ligand for the targeted enzyme [73,79]. Galantamine achieved a docking score of −7.8 kcal/mol. Therefore, ligands with docking energies close to this value are likely to have high binding affinities with the targeted receptor. As shown in Table 4, the majority of the studied compounds can be considered as potential inhibitors of AChE.

The interaction profiles analysis of several docked constituents reveals numerous similarities with that of galantamine. The galantamine molecule forms four hydrogen bonds with three different residues, GLY:172, TYR:184, and SER:259, carbon hydrogen bond with TYR:391, π-cation bond with HIS:502, π-π T-shaped bonds with TYR:391 and PHE:392, and four π-alkyl bonds with TRP:126, TYR:391, and HIS:502. Interestingly, although their binding energies are slightly higher than that of galantamine, elemyl acetate (**27**) and (*E*,*E*) farnesyl acetate (**33**) exhibit better affinity within the active site of AChE based on the number of interactions and amino acids they engage with. The presence of the ester functional group in their chemical structures may explain their specific affinities in the active site [81,82]. The oxygenated sesquiterpene (**27**) (−6.6 kcal/mol) is implicated in twenty-one interactions with nine amino acids. It formed three hydrogen bonds with GLY:172, GLY:173, and SER:259, a carbon hydrogen bond with GLY:172, and seventeen π-alkyl bonds with TYR:114, TRP:126, PHE:351, TYR:391, PHE:392 and HIS:502. Furthermore, the interactions profile of the oxygenated sesquiterpene (**33**) (−7.5 kcal/mol), which displays the most impressive binding energy among the tested ligands, reveals that it forms fourteen non-covalent interactions with nine different residues. Two hydrogen bonds with TYR:391 and TYR:395 were detected, with a carbon hydrogen bond with GLU:123, an alkyl bond with MET:501, and ten π-alkyl bonds with TYR:114, TRP:126, PHE:351, TYR:391, PHE:392, TYR:395, and HIS:502.

The rest of the studied components were arranged as follows: elemol, (*E*)-β-caryophyllene, α-eudesmol, caryophyllene oxide, β-eudesmol, and germacrene B with Autodock Vina binding energies of −6.9, −6.6, −6.2, −6.1, −5.9, and −5.7 kcal/mol, respectively. A series of hydrophobic interactions were also noted for each compound. Compared to galantamine, the TYR:126 amino acid is the common residue shared by all docked compounds, alongside HIS:502 residue, which is absent only in caryophyllene oxide.

The molecular docking results, including optimal ligands positions, low binding energies values, and the high number of interactions in the active site of the target enzyme, effectively demonstrate the potent anti-acetylcholinesterase activity exhibited by crude REO and especially its fractions.

### 3.5. DFT Study

**Figure 5:**
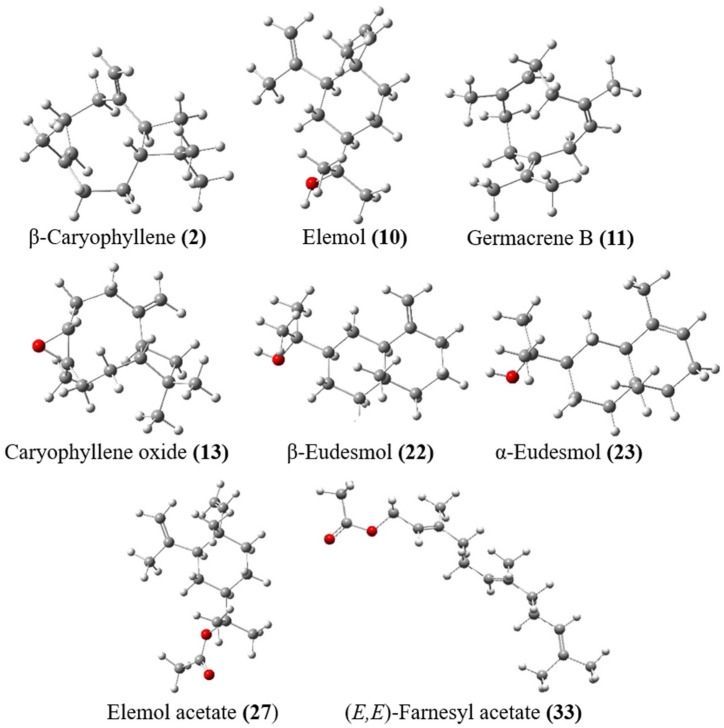
Optimized structure of the major compounds identified in resin essential oil of *Schinus molle* and its fractions using the DFT/6-31(G) (d, p) basis set.

The main selected compounds from EO and F_1–3_ were entirely optimized using the DFT/B3LYP method with “6-31(G)” basis set as illustrated in Figure 5. Table 5 presents the calculated quantum chemical properties of these compounds employed to optimize their molecular structures, which include electron affinity (EA), ionization potential (IP), energy gap (ΔE_gap_), chemical potential (μ), global hardness (ɳ), global softness (*S*), electronegativity (χ), global electrophilicity index (ω), dipole moment (Dm), and electronic charge (ΔN_max_).

As they are directly proportional to each other, the dipole moments and the chemical reactivities of the molecules can be correlated [83]. (*E*,*E*)-farnesyl acetate (**33**) exhibited the highest dipole moment (4.681 Debye), followed by elemol (**10**) with Dm = 2.051 Debye, whereas (*E*)-β-caryophyllene (**2**) showed the lowest one (0.354 Debye). Table 5 shows that in complete contrast to the properties of (*E*)-β-caryophyllene (ɳ = 3.232 eV; *S* = 0.155 eV), β-eudesmol (**22**) has the highest hardness of 3.590 eV and the lowest softness of 0.139 eV, implying its classification as the least reactive system.

**Figure 6:**
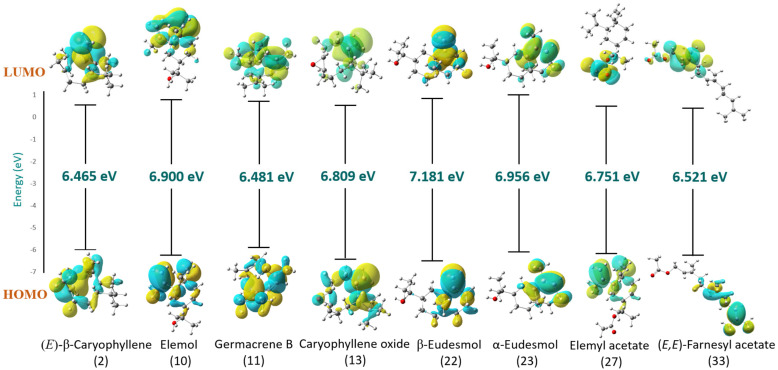
DFT-based HOMO and LUMO Frontier Molecular Orbitals (FMO) of the main compounds identified in the resin essential oil of *Schinus molle* tree and its fractions (F_1–3_) using B3LYP/6-31G (d, p) method.

“Frontier Molecular Orbitals (FMO)” is the common term that combines the two orbitals HOMO “Highest Occupied Molecular Orbital” and LUMO “Lowest Unoccupied Molecular Orbital”. The former acts as an electron donor, while the latter serves as an electron acceptor. These two specific orbitals play a crucial role in determining chemical reactivity and stability [84]. Table 5 displays the energy values of HOMO and LUMO orbitals for each constituent. Additionally, their energy differences (ΔE_gap_) and the corresponding electron density maps are displayed in Figure 6, highlighting the specific regions within each molecule where these orbitals are localized. The blue and yellow colors represent the positive and negative phases, respectively. As seen, the energy gap values are quite similar; however, the order of the ΔE_gap_ (energy gap) was as follows; (*E*)-β-caryophyllene < germacrene B < (*E*,*E*)-farnesyl acetate < elemyl acetate < caryophyllene oxide < elemol < α-eudesmol < β-eudesmol. A larger energy gap between the HOMO and LUMO orbitals of a molecule indicates a more stable electronic configuration, making it less susceptible to excitation. Consequently, the molecule exhibits lower reactivity. Therefore, when compared to the other compounds, β-eudesmol (**22**) exhibits the largest ΔE_gap_, suggesting its high kinetic stability. However, (*E*)-β-caryophyllene (**2**) demonstrates the lowest ΔE_gap_, suggesting its higher chemical reactivity. For (*E*)-β-caryophyllene (**2**) and germacrene B (**11**), the ionization energy (potential) was the lowest, revealing that these phytochemicals are the best donors of electron density. Meanwhile, for elemol (**10**), β-eudesmol (**22**), and α-eudesmol (**23**), their electron affinity (EA) values were more negative compared to the rest of the studied compounds, suggesting that they would hardly receive additional electrons. Moreover, it is worth mentioning that the highest value of ω (global electrophilicity) was obtained for (*E*,*E*)-farnesyl acetate (**33**); compared to the other compounds, this reveals that this compound is more capable of generating substantial interactions with biological macromolecules such as enzymes [85]. This is consistent with the results of the molecular docking study, in which this compound showed the lowest binding energy value (−7.5 kcal/mol) with good affinity for the AChE enzyme.

**Figure 7:**
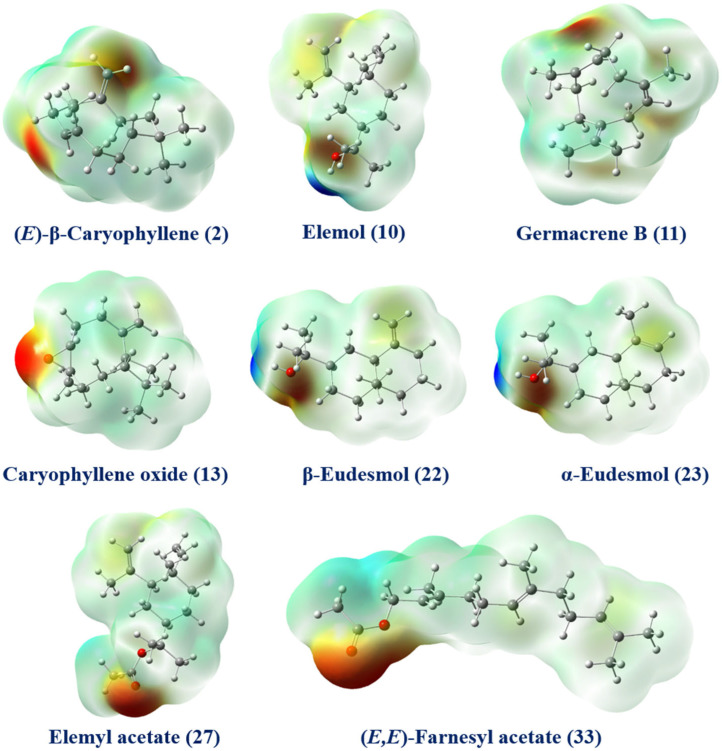
3D Molecular electrostatic potential (MEP) surfaces of the major compounds identified in resin essential oil of *Schinus molle* and its fractions (F_1–3_). The red regions correspond to negative potentials and the blue regions to positive potentials.

The MEP (Molecular Electrostatic Potential) map is an effective tool for predicting a molecule’s reactive regions for electrophilic and nucleophilic interactions, as well as for biological recognition [86]. The MEP diagram offers a visual representation of electrostatic potential (EP) regions, using color-coding to distinguish between negative, positive, and neutral areas. The blue areas on the MEP diagram represent regions with intensely positive (+) EP, while the red areas indicate zones with intensely negative (−) EP. Green areas depict regions with neutral potential. The blue regions (positive potential) are associated with nucleophilic attacks, and the red regions (negative potential) are linked to electrophilic attacks. Using the DFT/B3LYP method at the 6-31G (d, p) level calculation, Figure 7 illustrates the MEP surfaces of the main compounds identified in resin essential oil of *S. molle* and its fractions (F_1–3_). For elemol (**10**), β-eudesmol (**22**), and α-eudesmol (**23**), their MEP surfaces showed negative areas (red) around the oxygen atoms of their hydroxyl (−OH) groups, while blue surfaces (positive region) appeared around their hydrogen atoms of the same group, making them good sites for nucleophilic attacks. Concerning (*E*)-β-caryophyllene (**2**) and germacrene B (**11**), the MEP model presented negative zones (red), susceptible to electrophilic attack around the double bonds (π). A high electron density was observed around the epoxy group of caryophyllene oxide (**13**). On the other hand, intense negative potentials are located mainly around the carbonyl oxygen atoms of elemyl acetate (**27**) and (*E*,*E*)-farnesyl acetate (**33**). Variations in the potential and charge distribution around these constitutions are likely the main factors influencing their different biological activities.

## 4. Conclusions

For the first time, the chemical profiles of Tunisian *S. molle* resin essential oil (REO) and its fractions were reported. Hidden compounds were identified in the complex chemical mixture of the REO through the chromatographic fractionation. In total, 33 constituents were identified in the resin. The essential oil was dominated by two classes of compounds: sesquiterpene hydrocarbons (nearly 50%) and oxygenated sesquiterpenes (48.4%). The insecticidal effectiveness of the essential oil (EO) and its three separate fractions was evaluated against *Tribolium castaneum* adults, a widespread pest known to infest stored food products. The essential oil displayed significant repellent, fumigant, and topical toxicity, even though the treatment time was short. More interestingly, the findings from the fractionated samples (F_1–3_) were noteworthy, supporting the significance of the employed fractionation method. This could be attributed to the potential concentration of specific bioactive compounds during the process. To better understand the mode of action of this oil, the studied REO and its fractions were tested for their acetylcholinesterase inhibition potential, and they showed interesting results. Encouraged by these findings, the main detected compounds of REO and its fractions were docked into the *T. castaneum* acetylcholinesterase enzyme, yielding promising results. In addition, a DFT study was performed to assess their reactivity and stability. The findings presented here offer a promising foundation for exploring the potential of *S. molle* plant material as a natural alternative to highly toxic chemical insecticides used by the agrochemical industry to control *T. castaneum* infestations.

## Figures and Tables

**Table 1:** Chemical composition of resin essential oil (REO) of *Schinus molle* and its fractions (F_1–3_).

				Composition (%)				
N°	Compounds	R.I ^a^	R.I ^b^	REO	F_1_	F_2_	F_3_	Identification
1	β-Elemene	1392	1389	2.0	2.4	-	-	GC–MS, RI
2	(*E*)-β-Caryophyllene	1419	1418	**17.7**	**24.2**	-	-	GC–MS, RI
3	γ-Elemene	1434	1434	2.4	2.9	-	-	GC–MS, RI
4	α-Humulene	1455	1452	1.7	2.2	-	-	GC–MS, RI
5	*epi*-Cubebol	1494	1493	3.5	5.7	-	2.3	GC–MS, RI
6	α-Selinene	1495	1498	2.4	3.2	-	-	GC–MS, RI
7	Cubebol	1515	1514	-	-	-	3.1	GC–MS, RI
8	Calamenene	1531	1529	0.9	1.2	-	-	GC–MS, RI
9	Selina-3(7),11-diene	1542	1545	1.4	2.3	-	-	GC–MS, RI
10	Elemol	1550	1548	3.6	1.8	-	**16.6**	GC–MS, RI
11	Germacrene B	1557	1559	**21.4**	**26.5**	2.6	-	GC–MS, RI
12	Germacrene D-4-ol	1575	1574	-	-	4.0	-	GC–MS, RI
13	Caryophyllene oxide	1581	1582	**15.1**	**21.7**	4.4	-	GC–MS, RI
14	Viridiflorol	1591	1592	0.8	-	-	3.7	GC–MS, RI
15	Humulene epoxide II	1607	1608	1.0	1.3	-	-	GC–MS, RI
16	Humulane-1,6-dien-3-ol	1619	1619	-	-	1.7	-	GC–MS, RI
17	1-*epi*-Cubenol	1629	1627	1.7	-	3.7	-	GC–MS, RI
18	γ-Eudesmol	1632	1630	2.5	-	-	6.6	GC–MS, RI
19	Caryophylla-4(12),8(13-dien-5-ol	1639	1639	-	0.9	-	-	GC–MS, RI
20	T-Cadinol	1641	1640	2.0	-	-	5.8	GC–MS, RI
21	Cubenol	1642	1645	1.9	-	2.8	-	GC–MS, RI
22	β-Eudesmol	1650	1649	5.5	-	-	**16.0**	GC–MS, RI
23	α-Eudesmol	1651	1652	3.6	-	-	**26.8**	GC–MS, RI
24	α-Cadinol	1652	1652	2.7	-	1.4	-	GC–MS, RI
25	*epi*-γ-Eudesmol	1663	1664	-	-	1.4	-	GC–MS, RI
26	Bulnesol	1667	1670	-	-	-	1.2	GC–MS, RI
27	Elemyl acetate	1680	1680	2.5	-	**10.9**	-	GC–MS, RI
28	(*Z*,*E*)-Farnesol	1691	1690	-	-	-	2.5	GC–MS, RI
29	Juniper camphor	1693	1693	-	-	2.6	6.6	GC–MS, RI
30	(*E*,*E*)-Farnesol	1723	1722	-	-	-	3.9	GC–MS, RI
31	β-Eudesmyl acetate	1790	1792	-	-	3.2	-	GC–MS, RI
32	γ-Eudesmyl acetate	1791	1794	-	-	6.6	-	GC–MS, RI
33	(*E*,*E*)-Farnesyl acetate	1843	1845	2.0	-	**48.3**	-	GC–MS, RI
	Monoterpene hydrocarbons			0.0	0.0	0.0	0.0	
	Oxygenated monoterpenes			0.0	0.0	0.0	0.0	
	Sesquiterpene hydrocarbons			49.9	64.9	2.6	0.0	
	Oxygenated sesquiterpenes			48.4	31.4	91.0	95.1	
	Non-terpene derivatives			0.0	0.0	0.0	0.0	
	**Total identified**			**98.3**	**96.3**	**93.6**	**95.1**	

**Table 2:** Repellent activity of resin essential oil (REO) of *Schinus molle* tree and its fractions (F_1–3_) on *Tribolium castaneum* adults.

Sample	Exposure Time (min)	Repellency (%)	Class	Effect
REO	15	50.0 ± 10.0 ^b^	III	Moderately repellent
	30	56.7 ± 5.8 ^d^	III	Moderately repellent
	60	60.0 ± 0.0 ^e^	IV	Repellent
	120	76.7 ± 5.8 ^g^	IV	Repellent
F_1_	15	53.3 ± 5.8 ^c^	III	Moderately repellent
	30	60.0 ± 0.0 ^d^	IV	Repellent
	60	76.7 ± 5.8 ^g^	IV	Repellent
	120	86.7 ± 5.8 ^h^	V	Very repellent
F_2_	15	46.7 ± 5.8 ^a^	III	Moderately repellent
	30	63.3 ± 5.8 ^e^	IV	Repellent
	60	70.0 ± 0.0 ^f^	IV	Repellent
	120	73.3 ± 5.8 ^fg^	IV	Repellent
F_3_	15	73.3 ± 5.8 ^fg^	IV	Repellent
	30	73.3 ± 5.8 ^fg^	IV	Repellent
	60	90.0 ± 10.0 ^h^	V	Very repellent
	120	96.7 ± 5.8 ^i^	V	Very repellent

Repellency (%) are listed as mean value ± standard error. The repellency was classifed according to the scale of McDonald composed of six repulsive classes; Class 0 (PR < 0.1): non repellent, class I (0.1% < PR ≤ 20%): very weakly repellent, class II (20.1% < PR ≤ 40%): weakly repellent, Class III (40.1% < PR ≤ 60%): moderately repellent, class IV (60.1% < PR ≤ 80%): repellent, class V (80.1% < PR ≤ 100%): very repellent. Statistically different mean values (*p* < 0.05) are represented by different letters in superscript.

**Table 3:** Anti-acetylcholinesterase activity of the resin essential oil (REO) of *Schinus molle* tree and its fractions (F_1–3_). The same letter in superscript indicates that there is no statistically significant difference at a *p*-value less than 0.05.

Sample	IC_50_ (μg/mL)
REO	88.55 ± 4.00 ^d^
F_1_	42.77 ± 2.99 ^c^
F_2_	15.25 ± 1.48 ^b^
F_3_	13.07 ± 1.22 ^a^

**Table 4:** Interaction details and docking scores of the main compounds (>10%) of resin essential oil from *Schinus molle* in the active pocket of the *Tribolium castaneum* acetylcholinesterase enzyme, as predicted by Autodock Vina software.

N°	Compound Name	Binding Energy (kcal/mol)	Interaction Detail: NI/NIAA: IAA
**2**	(*E*)-β-Caryophyllene	−6.6	12/4: TYR:114, TRP:126, TYR:391, HIS:502
**10**	Elemol	−6.9	12/5: TYR:114 *, TRP:126, PHE:351, TYR:391, HIS:502
**11**	Germacrene B	−5.7	6/4: TYR:114, TRP:126, TYR:391, HIS:502
**13**	Caryophyllene oxide	−6.1	12/4: MET:125, TRP:126, ILE:183, LEU:493 *
**22**	β-Eudesmol	−5.9	11/4: TYR:114, TRP:126, TYR:391*, HIS:502
**23**	α-Eudesmol	−6.2	9/5: TYR:114, TRP:126, TYR:184 *, TYR:391, HIS:502
**27**	Elemyl acetate	−6.6	21/9: TYR:114, TRP:126, GLY:172 *, GLY:173 *, SER:259 *, PHE:351, TYR:391, PHE:392, HIS:502
**33**	(*E*,*E*)-Farnesyl acetate	−7.5	14/9: TYR:114, GLU:123, TRP:126, PHE:351, TYR:391 *, PHE:392, TYR:395 *, MET:501, HIS:502
Galantamine		−7.8	12/7: TRP:126, GLY:172 *, TYR:184 *, SER:259 **, TYR:391, PHE:392, HIS:502

*: 1 hydrogen bond, **: 2 hydrogen bonds, NI: Number of interactions, NIAA: Number of interacting amino acids, IAA: Interacting amino acids.

**Table 5:** DFT-based values of chemical descriptors of the main compounds identified in the resin essential oil of *Schinus molle* tree and its fractions (F_1–3_) using B3LYP/6-31G (d, p) method.

Comp.	E_HOMO_ (eV)	E_LUMO_ (eV)	EA (eV)	IP (eV)	ΔE_gap_ (eV)	μ (eV)	ɳ (eV)	S (eV)	ꭓ (eV)	ω (eV)	Dm (D)	ΔN_max_
(*E*)-β-Caryophyllene (**2**)	−5.954	0.510	−0.510	5.954	6.465	−2.722	3.232	0.155	2.722	1.146	0.354	0.842
Elemol (**10**)	−6.189	0.712	−0.712	6.189	6.900	−2.739	3.450	0.145	2.739	1.087	2.051	0.794
Germacrene B (**11**)	−5.829	0.653	−0.653	5.829	6.481	−2.588	3.241	0.154	2.588	1.033	0.551	0.799
Caryophyllene oxide (**13**)	−6.360	0.449	−0.449	6.360	6.809	−2.956	3.404	0.147	2.956	1.283	1.767	0.868
β-Eudesmol (**22**)	−6.422	0.759	−0.759	6.422	7.181	−2.832	3.590	0.139	2.832	1.117	0.936	0.789
α-Eudesmol (**23**)	−6.040	0.916	−0.916	6.040	6.956	−2.562	3.478	0.144	2.562	0.944	1.438	0.737
Elemyl acetate (**27**)	−6.342	0.409	−0.409	6.342	6.751	−2.967	3.375	0.148	2.967	1.304	1.385	0.879
(*E*,*E*)-Farnesyl acetate (**33**)	−6.193	0.328	−0.328	6.193	6.521	−2.933	3.260	0.153	2.933	1.319	4.681	0.900

**E_HOMO_**: Energy of the Highest Occupied Molecular Orbital, **E_LUMO_**: Energy of the Lowest Unoccupied Molecular Orbital, EA = Electron Affinity, IP = Ionization Potential, ΔE_gap_ = Energy Gap, μ = Chemical Potential, ɳ = Hardness, S = Softness, χ = Electronegativity, ω = Global Electrophilicity Index, Dm = Dipole Moment, ΔN_max_ = Electronic Charge.

## Data Availability

Data is contained within the article.
